# C9orf72 deficiency contributes to synaptic hyperexcitability and excitotoxicity in frontotemporal dementia through CP‐AMPAR dysregulation

**DOI:** 10.1002/alz70855_102329

**Published:** 2025-12-23

**Authors:** Belay Gebregergis, Liam T Ralph, Liang Zhang, Graham L Collingridge, Janice Robertson

**Affiliations:** ^1^ University of Toronto, Toronto, ON, Canada; ^2^ Tanz Centre for Research in Neurodegenerative Diseases, Toronto, ON, Canada; ^3^ UHN, Toronto, ON, Canada; ^4^ Lunenfeld‐Tanenbaum Research Institute, Toronto, ON, Canada

## Abstract

**Background:**

Hexanucleotide repeat expansions in *C9orf72*, the most common genetic cause of frontotemporal dementia (FTD) and amyotrophic lateral sclerosis (ALS), are associated with haploinsufficiency, synaptic dysfunction, and neurodegeneration. Synaptic hyperexcitability and excitotoxicity are emerging hallmarks in *C9orf72*‐associated neurodegeneration; however, the underlying mechanisms in dementia‐related pathology remain poorly understood.

**Method:**

Using *C9orf72*‐knockout (C9‐KO) mice as a model, we investigated the role of C9orf72 in synaptic function and excitotoxic vulnerability. We assessed hippocampal synaptic markers, including GluA1 surface expression, dendritic spine morphology, and calcium‐permeable AMPA receptor (CP‐AMPAR)‐mediated plasticity. Kainic acid (KA)‐induced excitotoxic stress was used to evaluate seizure susceptibility, network stability via EEG analysis, and hippocampal GluA1 dysregulation. CP‐AMPAR antagonists were applied to examine their therapeutic potential.

**Result:**

C9‐KO mice exhibited enhanced synaptic hyperexcitability, characterized by elevated surface GluA1 expression, reduced dendritic spine density, and enlarged spine heads in hippocampal neurons. These changes correlated with enhanced CP‐AMPAR‐mediated synaptic plasticity and heightened excitotoxic vulnerability following KA treatment. C9‐KO mice displayed more severe seizures, abnormal EEG spectral power, and persistent hippocampal GluA1 elevation. Selective CP‐AMPAR antagonism effectively reduced excitotoxic damage and normalized synaptic function.

**Conclusion:**

Our findings demonstrate that *C9orf72* deficiency drives synaptic hyperexcitability and excitotoxic vulnerability through CP‐AMPAR dysregulation, implicating this pathway in the pathophysiology of FTD. At the network level, *C9orf72* loss amplifies excitatory signaling, linking synaptic dysfunction to network instability and neuronal degeneration. By identifying CP‐AMPARs as central mediators of excitotoxicity, this study highlights a novel therapeutic target for *C9orf72*‐associated dementia and other neurodegenerative diseases characterized by excitatory network dysfunction.

